# Objective Supervised Machine Learning-Based Classification and Inference of Biological Neuronal Networks

**DOI:** 10.3390/molecules27196256

**Published:** 2022-09-23

**Authors:** Michael Taynnan Barros, Harun Siljak, Peter Mullen, Constantinos Papadias, Jari Hyttinen, Nicola Marchetti

**Affiliations:** 1 Computational Biophysics and Imaging Group/BioMediTech, Faculty of Medicine and Health Technology, Tampere University, 33100 Tampere, Finland; 2 School of Computer Science and Electronic Engineering, University of Essex, Colchester CO4 3SQ, UK; 3 School of Engineering, Trinity College Dublin, D02 PN40 Dublin, Ireland; 4 American College of Greece, 153 42 Agia Paraskevi, Greece

**Keywords:** cortical circuits, neuroinformatics, supervised machine learning, cell-classification, network tomography, information theory

## Abstract

The classification of biological neuron types and networks poses challenges to the full understanding of the human brain’s organisation and functioning. In this paper, we develop a novel objective classification model of biological neuronal morphology and electrical types and their networks, based on the attributes of neuronal communication using supervised machine learning solutions. This presents advantages compared to the existing approaches in neuroinformatics since the data related to mutual information or delay between neurons obtained from spike trains are more abundant than conventional morphological data. We constructed two open-access computational platforms of various neuronal circuits from the Blue Brain Project realistic models, named Neurpy and Neurgen. Then, we investigated how we could perform network tomography with cortical neuronal circuits for the morphological, topological and electrical classification of neurons. We extracted the simulated data of 10,000 network topology combinations with five layers, 25 morphological type (m-type) cells, and 14 electrical type (e-type) cells. We applied the data to several different classifiers (including Support Vector Machine (SVM), Decision Trees, Random Forest, and Artificial Neural Networks). We achieved accuracies of up to 70%, and the inference of biological network structures using network tomography reached up to 65% of accuracy. Objective classification of biological networks can be achieved with cascaded machine learning methods using neuron communication data. SVM methods seem to perform better amongst used techniques. Our research not only contributes to existing classification efforts but sets the road-map for future usage of brain–machine interfaces towards an in vivo objective classification of neurons as a sensing mechanism of the brain’s structure.

## 1. Introduction

The detailed characterisation of the human brain has recently attracted investments in many countries, in both academia and industry, to help create a digital reconstruction of the brain and also move towards a complete understanding of its mysteries [1]. The majority of efforts are focusing on the issue of characterising morphological and electrical types of neurons for linking cellular activity to the whole functioning, behaviour and pathology characterisation of the human brain. Without solving this issue, the neuroscience community will keep basing its analysis on the unverified subjective classification of neurons, which is far from a precise approach [2,3]. In addition, this type of information is crucial for precise micro-scale brain–machine interfaces that can interact at the cellular level [4,5].

Despite the many anatomical studies, the recurring issue of classifying morphological neuron structures is about the lack of reliable results using methods of visual inspection of brain slices [6]. Morphological data consist of the geometrical neuron branch structures that define whether each cell has a centric or sparse distribution of connections, which translates to the overall number of synapses as well as the micro-space the neurons would reach. By using objective methods adopting neuroinformatics tools, we can reduce bias in the classification with digital solutions. The key benefit is thus a rigorous analysis of the neuronal data in order to provide verifiable and reproducible neuron classification. Recently proposed methods include supervised machine learning techniques based on cellular morphological structure data [2,3]. Compared to its visualisation-based approach counterpart, this technique is more efficient where non-trivial details in cell morphology are now captured with a machine learning solution. Even though this and other new approaches are exciting, we hypothesise that we can perform even more reliable objective morphological classification when including also cellular activity and communication data, based on the relationship between cellular morphology and activity, by using machine learning-based solutions [7]. The main added value of objective classification is the possibility of automation, and therefore by possibly applying these tools to electrophysiological signals of population of neurons in-vitro, in-vivo or ex-vivo, we will have the capacity to classify parts or the whole neuron types in those with a very quick turnaround time. This would take years of work to be done with classical methods. In this way, we would provide advantages as compared to the existing approaches, where, for instance, activity and communication are more accessible and abundant in data than morphology, and capture relationships not previously modelled. In addition, we can investigate how to use morphology–activity dynamics to infer larger structures than cells, e.g., networks.

Several brain–machine interfaces have been proposed in recent years which aim to offer measurement of cortical information through minimally invasive means. With the introduction of such data measurement techniques comes the need to further our understanding of the circuit-level characteristics of these neuronal systems such that the available data can be adequately utilised. Brain–machine interfaces will allow access to information of neuronal networks at a level similar to that available from the analysis of man-made network systems, bridging the gap between biological neuronal analysis and classical communication theory that has been developed for (and applied to) these existing network systems such as those discussed in [8,9].

**Figure 1:**
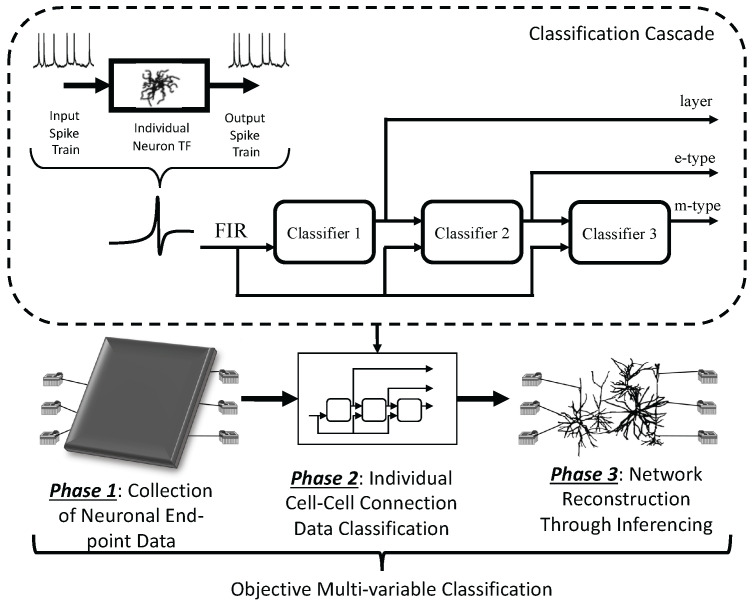
Overview of the objective multi-variable classification and inference of biological neuronal networks. First, in Phase 1, we collect end-point neuronal voltage data and this is performed through simulations of cortical neuron networks. We derive finite impulse responses (FIR) models from individual neuron transfer functions (TF) which are used in our mutual information and spike delay estimation analysis. Then, in Phases 2 and 3, we propose a cascade of supervised classification algorithms using features from our information and communication analysis based on various forms of cortical networks, from generic networks motifs to existing ones found in biology. Finally, we reconstructed the cell-types in a known topology based on end-point measurements taken around the network.

In this paper, *we propose a new objective multi-variate classification based on the morphology, activity and communication of neurons using supervised machine learning algorithms.* The main strengths of our work include (i) *the characterisation of neuronal information transfer using classical Shannon mutual information theory and spike delay estimation*, and (ii) *a cascade of three different classifiers arranged to perform classification and inference of biological neuronal networks using multiple variables with the same training data.* We depict our proposed system in Figure 1. First, in Phase 1, we collect end-point neuronal voltage data through simulations of cortical neuron networks. For that, *we developed new simulation support libraries for integrating large cortical circuit formation, using the Python language to link the validated computational models of the Blue Brain Project and the NEURON tool, called Neurpy and NeurGen.* We derive finite impulse responses (FIR) models from individual neuron transfer functions (TF) used in our mutual information and spike delay estimation analysis. In practice, we can assume that the measured signals are available (as they can, e.g., be measured by multi-electrode array technology in-vitro or by invasive brain–computer interfaces in-vivo), even though its utility in those scenarios must be verified by further studies. *Then, in Phases 2 and 3, we propose a cascade of supervised classification algorithms using features from our information and communication analysis based on various forms of cortical networks, from generic networks motifs to existing ones found in biology, as discussed in [10].* We investigate the classification and inference of neuronal cells and circuits, especially at an inter-cortical layer scale and with a large variety of cell-types (40+), progressing the field towards the classification of more complex cortical circuits. For that, we extracted the simulated data of 10,000 network topology combinations with five layers, 25 cell m-types, and 14 cell e-types. We applied the data to a cascade of different classifiers (including Support Vector Machine (SVM), Decision Trees, Random Forest, and Artificial Neural Networks) analysing accuracy, recall, precision and F1 score, and also identifying the specific cell type (and sub-group types). The classification and the inference are performed based on the conventional training/testing data split method (70–30 ratio), where the testing data are compared to the training data. Finally, we reconstructed the cell-types in a known topology based on end-point measurements taken around the network. We achieved accuracies of up to 70% and inference of biological network structures using network tomography reached up to 65% of accuracy.

The contributions of our work are as follows: (i) **A simulation support library referred to as** ***Neurpy***, which allows for the rapid generation, construction, and simulation of cortical networks. This library is written in Python and uses the NEURON-backend API while accepting XML-based network descriptors to define the cortical circuit. (ii) **A network-generation script termed** ***NeurGen***, which uses statistical information of neuronal pathways to generate large quantities of unique networks. The generated networks can be either random in shape or can be specified to fit a given topology template. The generated network descriptor is in a format which allows seamless integration with the *Neurpy* library. (iii) **The usage of discrete-memoryless mutual information between two cells for the characterisation of the intercellular information transfer.** We describe the process of train-discretisation for the conversion from a set of analogue voltage measurements into a binary sequence. This binary sequence is then used to calculate the discrete-memoryless mutual information between two cells. We also describe the process of simple delay-estimation using the cross-correlation of the network measurements, selecting peaks in the correlated result to predict the synaptic delay. (iv) **Classification and inference of biological neural network morphology and topology based on information network tomography.** We propose and implement a method of cell-characterisation through filter coefficient estimation using the pseudoinverse method for resolving linear equations, similar to existing cell-description models such as the *Linear-Nonlinear-Poisson* cascade model. Using this cell-characterisation, we describe the training process of many cell-type classification models to predict cell sub-groups from voltage measurements. Finally, we describe the process of topology-reconstruction using the supervised classification models to estimate the cell-type in a 4-leaf star topology. Three different classification algorithms are used independently to estimate the neuron layer, its electrical type and its morphological type for each node in the network.

### Related Work

We notice an increased interest about the problem of neural network topology discovery with different ways and variations of its definition, which shows that this exciting area is emerging and worth exploring in the future. For example, Ref. [11] explores artificial neuron networks, which makes the problem of network topology inferencing easier. Biological neuron networks have intrinsic characteristics that affect their topological classification due to their natural dynamical behaviour. In addition, that is somewhat the direction that the [12] is taking; however, since their level of generality is high, many dynamics captured by their model directly applicable to biological models. For our problem, biological neuron networks have a high level of biological plausibility and that is why we focus in acquiring data and models from the Blue Brain Project. Ref. [13] explores the same problem as ours but using another data type, imaging, which can impose many benefits as direct visualisation of connections. However, the need of identifying the neuron types as well is important for uncovering the true functionality of the network, and the authors do not explore such problems and only focus the discovery of the connectivity of the network. The investigation of the information transfer in neuron communications is an active research topic in the field of molecular communications, aiming to further understand biological signal propagation [14]. Even though researchers have focused on the molecular synaptic transfer [15], or single-cell models [16], works such as [17,18] progressed towards neuron population scenarios to analyse more impactful information transfer in the brain. Prior work investigating the mutual information in neural communications presented in [19] has expressed the entropy of a source through the time-binning of the spike train, and defining the set of possible symbols as the possible neural response within the period. Other methods were based on type-rich network structures but suggested that network connections are more relevant to the information transfer inside a network than the main cellular differences between types [17]. The main issue presented in their research is that a large data set is required to converge to a true entropy value. We aim to instead calculate a lower and upper bound on the entropy value which are robust regardless of the dataset length. Works like [2,3,6] have provided many contributions towards the objective classification of neurons, by analysing morphological structures data using Bayesian analysis and clustering algorithms. The major criticism is that (i) they base their classification method solely on morphology and (ii) morphological training data are difficult to accumulate, and this limits the creation of adaptable classification techniques [20]. Morphological data also limit the usage of existing approaches for more complex structures, including cortical microcircuits. Our objective is to make objective neuron classification expand in different ways: (i) we aim to make it more reliable, (ii) make it useful for other higher complexity neuron structures and networks and (iii) provide a method that is more accessible for other researchers in the area or those dependent on these classification methods for other tasks. Recently, Ref. [4] investigated the classification of cortical network parameters given simulated end-point data using a theory called *Network Tomography*. A single post-synaptic cell-type, and four different combinations of pre-synaptic cell types, were selected. The post-synaptic cell was then stimulated, and the soma-membrane voltage was measured for each of the pre-synaptic groups. The features used in this classification model were the frequency, delay, and number of spikes, along with the voltage-vs.-time measurement of soma-membrane potential, and the average power spectral density. The overall accuracy of this classification model was about 83%, with the model performing well for four different combinations of four pre-synaptic groups. As well as cell-group classification, a topology–discovery classifier was also investigated in that work. The topologies investigated were relatively small and the classifier achieved accuracy as high as 99.37% when classifying the 2-leaf and 4-leaf topologies through the use of decision trees using the same cell types. While the research conducted focused mainly on a relatively small number of same type neurons, we expand on this by investigating similar types of classification with more complex sets of classes towards more realistic cortical structures, such as with the full set of cell-possibilities, which was completely abstracted in the work of [4]. There are many differences between this work and the one previously mentioned, as now we developed a proper classification tool that takes not only a simple network as an example but thousands, which is translated into the complete evaluation of the conceptual work. Moreover, and at a more detailed level, our findings here show a set of features to be used in machine learning solutions for the problem of objective classification. This is a more profound contribution, since it allows an increase of performance and the roadmap to improve it further. Of course, that depended on our contributions for estimating communication metrics such as delay and mutual information, but the trend is that modification of the estimators or new communication metrics will lead to better classification results.

## 2. Methods

**Figure 2:**
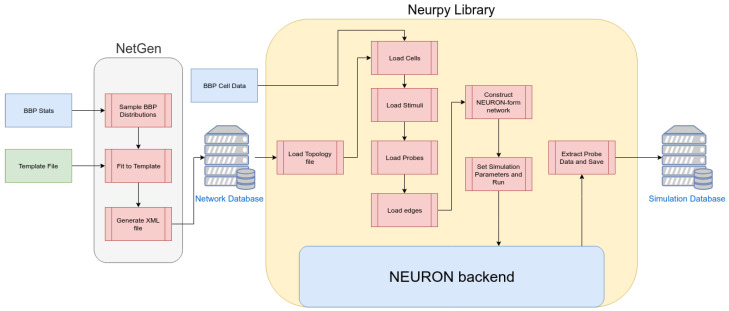
Structure of experimental environment: network (topology) generation, cell information input and simulation.

Our methodological framework is composed of the NetGen tool, which comprises the generation of network, the Neurpy library, which will simulate the network using a NEURON backend. Simulations will be used to generate training data for our machine learning classification methods. Figure 2 shows the overview of our methodology, which will be detailed described in the following.

### 2.1. Cortical Networks Characterisation

In this work, we focus on the neurons of the somatosensory cortex of a juvenile rat. The neocortex is typically treated as being a tightly-packed system of vertical structures (cortical columns) and classified into six interconnected horizontal layers, referred to as L1–L6 (with layers 2 and 3 often grouped and referred to as L2/3). Neurons can be classified by m-type, which defines the physical shape and layout of the neuron, and by e-type which defines the electrical characteristics of the cell. The neurons in this investigation are based on data retrieved from a single cortical column, where each neuron is pre-defined by layer, m-type, and e-type.

#### 2.1.1. Cell Characterisation

We focus our modelling on the characterisation of the response of the many neurons m-types to a given stimulus signal. This response is an *all-or-none* spike train response. We do not explore the description of the biophysical model of neurons in the paper since we believe that information is covered by the NEURON framework and the Blue Brain Project. Rather, we focus on the analysis of each neuron as a high-level system. As the release of neurotransmitters in the synapse is a response to a spike event rather than to the small variations in resting potential, the response of a postsynaptic cell is therefore also a function of the spike events. Analytically, the spike events are treated in a probabilistic fashion (i.e., the probability of a spike occurring) to which a Poisson process fits well [21]. The Poisson process describes a model for the estimated time between events, and so when applied in this domain to estimate the time between voltage spikes, the intercellular signals are seen as a *Poisson Spike Train*. This has several useful implications for the analysis of these intercellular signals, since the probabilistic modelling of the signal allows for the application of the Poisson model in information and communication domains that rely on signal probabilities, such as mutual information and delay. Another implication of this spike-triggered-event property is that the cell can be described as a time-varying process responding to (and with) an impulse train. One such model commonly used is the *Linear-Nonlinear-Poisson cascade* (LNP) model discussed in [22] where the response of a cell is described by three serial components: a linear filter, a nonlinear transform, and a Poisson spike-generating process. In the LNP model, the dimensionality reduction occurs in the linear-filter component (the nonlinearity and Poisson component deal mainly with the production of a spike train which is not required here). While this model generally deals with a spatio-temporal input (i.e., an external screen), we can adapt the concept to use the output spike-train of another neuron as input. As the spike-train is essentially an impulse-train, the resulting linear filter that best characterises the neuron can be estimated as the impulse response of a time-varying system taking an impulse train as input and generating a continuous voltage-time output. In our case, a finite impulse response (FIR) filter model was used. By measuring the input and output spike trains of a single cell, an estimation can be determined of the k-order FIR filter that best characterises the neuron’s impulse response [23,24]. The theoretical principle here is that, given the linear system y=x⋆h, one needs to estimate the value of *h* to a limited order that best produces the output sequence *y* from input sequence *x* (where ⋆ denotes the convolution operation). If we take this to be representative of a finite-impulse response (FIR) filter, we can describe the system as $\vec{y}=X\vec{h}$, where $\vec{h}$ is a vector of the coefficients of a *K*-order FIR filter, $\vec{y}$ is the vector of *N* output measurements, and X is an N×K matrix representing the time-shifted window of input measurements, through *N* time-shifts and a window size of *K*. This system is not invertible to solve for $\vec{h}$ where X is not square; however, it is possible to solve for *h* under “general conditions” through the use of the *pseudoinverse* of X. This can be more specifically defined by the Moore–Penrose pseudoinverse of X, so $\vec{h}$ is given by

(1) $$\vec{h}={\left(X^{T}X\right)}^{-1}X^{T}\vec{y},$$

where $\vec{y}$ is the output signal vector.

#### 2.1.2. Train Discretisation and Probability Analysis

**Figure 3:**
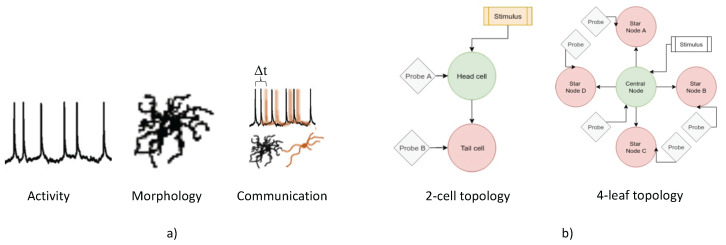
The visual representation of the (**a**) data types used in our paper (activity, morphology and communication) and (**b**) the topological configurations (2-cell topology and 4-leaf topology).

The data types used to account for the classification features are the neuron activity, morphology and communications, as they are depicted in Figure 3. The first step in the probabilistic analysis is to discretise the voltage-time spike trains into a binary series (activity), similar to the process followed in [19]. This approach is reasonable from a computing cost perspective, where the spike trains discretization process allows straighforward development of signal metric estimators to be used in our classifiers. Considering analogue neural transmission instead, one must account not only for entire action potentials, sub-threshold signals, ion channels around the membrane as well as synapses [25], which makes the cost of improving overall performance possibly not suitable for classification problems. In our case, the process undertaken was as follows: (i) Threshold the spike-train—values above the voltage threshold $V_{thr}$ are set to 1, all other values are set to 0; (ii) Subdivide the thresholded train into many windows of length $L_{w}$; (iii) Check for the presence of a spike within the window, set the window-value to 1 if found, 0 otherwise; (iv) Convert the window-interval values into a binary sequence.

The binary output sequence of this process represents the symbols of the system; in this case, our symbol is 1 bit and so our symbol set can be described as $S=\{s_{1}=0,s_{2}=1\}$. Here, we can calculate the binary sequence from the input, the head-cell (which we can represent as a random variable *X*) as well as from the output, the tail-cell (represented as random variable *Y*). The head-cell is the central node, which has connections to all other nodes in the network. The tail-cell corresponds to the edge nodes, which are connecting to the head-cell. A head-cell is the neuron with the highest connection degree inside the network, and the tail-cell is the one with the lowest. With this in mind, we can use the entropy model of the head-cell and mutual information of the cortical link, which is presented in the following. First, we calculate the probability mass function (PMF) of both X and Y (that is, P(X) and P(Y)) from their respective binary sequences. Then, we apply various forms of Bayes’ rule, along with further analysis of the two binary sequences, to obtain the joint PMF (P(X,Y)) and the conditional PMF (P(X|Y)) for the calculation of the mutual information between a head-cell and a tail-cell.

#### 2.1.3. Cortical Topologies

There are two main topologies investigated in this study: 2-cell topology, and 4-leaf star topology. The 2-cell topology consists of two cells linked by a synaptic connection whose parameters vary from network to network. It is a simple topology used as a starting point in this study mainly to analyse the link between two neurons rather than to investigate the various effects of higher-complexity neuronal layouts. The 4-leaf star topology is only a slightly more complex network, but enough to allow for richer behaviour. The use of this network intends to analyse the application of the results found from the study of the 2-cell networks in more complex topologies to investigate what effects, if any, the topology may have on performance. It is important to note here that the direction of the synaptic connections is that of an “out-hub”, i.e., the central node (acting as the hub) is delivering voltage spikes to the surrounding leaves. The main reason for starting with these topologies’ size is that they mimic the small-world topology, which is believed to be the underlying structure of complex networks in the brain [26]. Other topologies include the link-radius networks, shortcut networks, mesh networks as well as more robust network topology dynamics including Erdős–Rényi networks, which is briefly discussed in [26].

### 2.2. Information Theoretic-Based Network Tomography Based on Spike Delay Estimation

Network tomography is a branch of information and communication theory that deals with the inference of internal network properties from a finite number of end-point measurements. This concept can be applied in many specific applications; however, it is generally used in internet systems to determine link-loss and link-delay characteristics as discussed in [27]. Analytically speaking, the problem of broad inference of the entire network can be approximated by describing the measurements as a linear model [27] given by $\vec{y}=A\vec{\theta }+\epsilon$, where $\vec{y}$ is a vector of measurements, A is a routing matrix representing the network node connectivity, $\vec{\theta }$ is a vector of link parameters (delay, loss, etc.), and ϵ is a noise vector. The routing network is typically a binary matrix with the (i,j)th element being 1 to represent a connection between the *i*th node and the *j*th node, and 0 representing no connection between the two nodes. The network inference, in this case, would be estimating the vector $\vec{\theta }$ given some end-point measurements $\vec{y}$ and knowledge of the networking routing matrix, along with some distribution for the noise parameter ϵ (Gaussian, Poisson, etc.). For large networks, this poses a problem in the computational solution for the linear model, as the dimensionality of A can grow prohibitively large for sizeable networks. It is worth noting that the usual networks subject to tomography (e.g., internet) are bi-directional. In cortical circuits, the cell-to-cell links are mostly unidirectional with signals propagating in a single direction. This has many implications for the routing matrix A, namely that the presence of a 1 for element $A_{i,j}$ implies a high probability of a 0 for element $A_{j,i}$.

We also observe our networks from the *information theoretic* standpoint. The information theory of discrete systems deals largely with symbols. As there is a close link between information theory and probability, a symbol can be thought of being similar to a discrete random variable, where a source may produce a symbol at every event from a set of possible symbol values. The information contained in a given symbol is defined by $I\left(s_{k}\right)=-\log_{2}\left(p_{k}\right)$ where $s_{k}$ belongs to the symbol set $\left\{s_{1},\ldots ,s_{N}\right\}$ and $p_{k}$ is the probability of the corresponding symbol occurring. By taking the *average* entropy of each symbol in a given set (weighted by symbol probability), we obtain the *entropy* of the set, defined as $H\left(X\right)=-\sum_{k=1}^{N}p\left(s_{k}\right)\log_{2}\left(p\left(s_{k}\right)\right)$, where H(X) is the expected uncertainty of event source *X*. Another useful metric in information theory is that of *conditional entropy* or the entropy of some random variable given the knowledge of another random variable. Given the random variable *X*, the entropy of *X* given a particular value of another random variable $Y=y_{k}$ is given by

(2) $$\begin{array}{cc} \; & H\left(X|Y\right)=\sum_{k=1}^{N}H\left(X|Y=y_{k}\right)p\left(y_{k}\right)\\ \; & =-\sum_{k=1}^{N}\sum_{j=1}^{N}p\left(x_{j},y_{k}\right)\log_{2}\left(p\left(x_{j}|y_{k}\right)\right), \end{array}$$

where H(X|Y) is the conditional entropy of *X* given *Y*, $p(x_{j},y_{k})$ is the joint probability of symbols $x_{j}$ and $y_{k}$, and $p(x_{j}|y_{k})$ is the conditional probability as $\frac{P\left(y_{k}|x_{j}\right)P\left(x_{j}\right)}{P(y_{k})}$. Given this definition of the conditional entropy, we can obtain an expression for the reduction in entropy of *X*, given some observed event *Y*. This is defined by

(3) I(X;Y)=H(X)−H(X|Y),

where I(X;Y) is referred to as the *mutual information* of *X* and *Y*.

#### Delay Estimation

Following the collection of the simulation data, our analysis concentrates on the estimation of the link delay through the use of the cross-correlation between the two measured voltage plots, $x_{n}$ for the head-cell and $y_{n}$ for the tail-cell where $n\in {ℜ}^{+}$. This was implemented by the cross-correlation of these two series. First, we define the true cross-correlation of $x_{n}$ and $y_{n}$ as

(4) $$R_{x,y}\left(m\right)=E\left[x_{n+m}y_{n}^{*}\right]=E\left[x_{n}^{*}y_{n+m}\right],$$

where the asterisk denotes the complex conjugation, and E[.] denotes the expected value operator. Since *n* is an infinite-length random variable, we can only estimate a real cross-correlation value. Therefore, we may define

(5) $$\hat{R}=\left\{\begin{array}{c} \sum_{n=0}^{N-m-1}x_{n+m}y_{n}^{*},m\geq 0\\ {\hat{R}}_{x,y}^{*}\left(-m\right),m<0, \end{array}\right.$$

which will lead to a final cross correlation of ${\hat{R}}_{x,y}^{*}\left(m-N\right)$ with m=1,2,…,2N−1.

Next, we find the closest positive peak in the cross-correlation using local maxima theorem in a series. We define the local maxima as the portions of the plot where the first derivative is 0 and the second derivative is negative (concave-down). By assuming that the first local maxima represent the delay, we can obtain a simple estimate of the link delay.

Conceptually, a spike in the head-cell should result in a spike in the tail-cell separated in time roughly by the delay as the spike crosses the synaptic connection. As a result, we should find a peak in the cross-correlation between the voltage measurements of the two cells as the time shift approaches the delay since at this time-shift the signals should be relatively well correlated. While this approach is quite basic and may not be overly accurate, it is a first step in expressing the characteristic function of the delay in the neuronal link, which is an important concept when applying network tomography to estimate the delay, as discussed in [28].

### 2.3. Neuronal Computational Framework

Here, we discuss the design and implementation of the simulation framework used to generate, construct, simulate, and analyse the neuronal circuits.

#### 2.3.1. The NEURON Tool

The NEURON tool is a simulation framework developed by researchers at Yale University for the in silico analysis of individual and networked neurons. The framework provides some tools for the construction, simulation, and recording of individual cell-models from biological principle building-blocks as well as networks built from individual neuronal models (see [29] for more details).

#### 2.3.2. Cell-Data Source

The cell data used by NEURON to load and simulate individual cells were provided by the Blue Brain Project (BBP) through the Neocortical Micro-Circuit (NMC) portal available at [30,31]. These data are supplied in a format that is compatible with the NEURON framework, including sample script files for initiating and simulating single-cell networks. Each cell supplied is categorised by the layer, m-type, e-type, and the variant number (multiple variants may exist for the same layer;m-type;e-type group). For example, the cell titled “L1_DAC_bNAC219_1” represents a layer 1 cell of DAC m-type and bNAC e-type. DAC is short for descending axonal collaterals and bNAC is short for burst non-adapting. For each cell, a number of data-files are supplied. One of the main files is the morphology descriptor. This file contains the complete description of the entire morphology of the cell, formatted as the NEURON-compatible section-segment hierarchy which can be loaded quickly into the framework. The supplied data describe the cell’s biophysical properties. These properties represent the specific electrical characteristics of various sections within the neuron and are vital to the accurate simulation of the cell.

#### 2.3.3. Python Support Library

While the sample files given by the BBP are extremely useful and informative, they are limited in that it is not inherently easy to connect multiple cells to form a multi-cellular network. Besides this, the use of the default GUI is not a scalable approach when generating a large set of training data. We therefore developed a Python library to address these issues. The library, referred to as *Neurpy* and available at [32] uses the NEURON-Python API to more easily construct simulations of any network form, while also allowing for either GUI interfacing (for interactive/debug purposes) as well as a more lightweight “headless” interface where the simulations are run entirely through the Python code without any need for user interaction. A network generator script named *NeurGen* was constructed in Python. NeurGen functions by loading the JSON-formatted statistical data on the physiology and anatomy of each cellular pathway, building an internal database of statistical distributions for each of the connective parameters, and then generating a network which conforms to the given distributions. For this study, we developed the option of using topology templates as well. The template defines the overall network shape (number of cells, inter-cell connections, the stimuli, and the probes) without requiring any particular data to be specified about any of the network components. Cell-type constraints are also accepted in the template, which limits the set of possible neural cells that can be selected for a given position in the topology. The complete code release is available at [33].

#### 2.3.4. Overview of Simulation Framework

At this point, all the components of the simulation framework have been defined. An overview of the entire process is shown in Figure 2, with the general flow of the process being from left to right. On the left, we begin with the network generator, where we feed the statistical data on the cell-to-cell pathways from the BBP along with a topology template into NeurGen. Within NeurGen, we can then generate a large number of unique individual networks, which we store as the ”Network Database”, a collection of XML files describing each network. After the database of networks has been created, we can begin feeding this into the Neurpy library, file by file. For each network file, Neurpy loads the specified cell model data from the BBP, loads and connects the required stimuli, loads and connects the required measuring probes, and finally connects the cells to form the network. Following this, the session simulation parameters are set (timestep, simulation length, etc.) and Neurpy instructs the NEURON backend to begin the simulation. After the simulation has completed, all data are extracted from the probe vectors, converted and concatenated in a Python-native format, before being written on the disk. By repeating this for all network files, we create the “Simulation Database” which contains the voltage-vs.-time data for each probe in each simulation, which we can then use for analysis.

The core intention of the study is to examine methods of characterising individual neurons in such a way that a classification model can be built on top of the characterisation. The individual neurons may appear in more complex networks; however, the technique is the main contribution as opposed to the analysis on a particular the network structure. In constructing a simulation environment to extract simulation data from a given cell-under-test, we stimulate the cell to elicit a response suitable for our analysis. We use the NEURON built-in stimulation sources, but these tend to be somewhat mechanical. While not invalid (in fact, results with the built-in stim sources may well be equivalent), the availability of the rest of the BBP dataset allowed the use of a signal source as a more fitting stimulation source to generate an input signal that would be more representative of the type of signal found in a wider network. The particular choice of signal source is not as important as its ability to elicit a suitable response from the cell-under-test. To generate the 2-cell network, we first choose the tail-cell at random. Once chosen, we find all potential head-cells that have a matching excitatory pathway for the tail-cell defined in the BBP dataset. From this list of potential head-cells, we choose the cell with the highest mean synapses-per-connection, as this should yield the the best response signal from the tail cell.

The NMC/BBP data provide a list of synapses for each cell. When connecting the cells together in Neurpy, we enumerate this list of synapses, filter out the inhibitory ones (as we only deal with excitatory connections), filter out non-dendritic synapse points, and then sort by facilitation relaxation time. We then select how many synapses we need from the list, and connect to them through NEURON. The same MOD files are used for each synapse, as provided by the NMC/BBP data; the synapse is defined by ProbAMPANMDA_EMS.mod. We do have multi-synaptic connections. The location of each synapse is defined in the list of synapses provided for the cells. As we can only analyse simulated data where the tail-cell has demonstrated a suitable response (i.e., has shown some form of output voltage correlated with the spike events of the presynaptic cell), we needed a relatively high number of synaptic connections. In the new simulations, the number of connections was based on sampling the distribution given in the NMC/BBP dataset multiplied by a factor of 4. The variation in synapse count is now less restrictive, and is based on a random sampling of the mean/std of synapse count as given by the base dataset. The sampling is positively biased (i.e., we sample only on the positive side of the mean) to prevent edge-cases where negative-synapse counts were sampled, and to prioritise usable data from the simulations.

#### 2.3.5. Simulation Dataset

The cortical networks for the study of the neuronal communication network parameters were generated using the NeurGen script described above. A simple 2-cell topology template was passed to the generator which defined a network of two cells (with no constraint on cell type), a stimulus on the head-cell, and a probe on each soma. As this portion of the investigation deals with the communication network parameters between any two cells in the cortical circuits, there was no need for a topology more complex than two cells. Consideration of more than two cell connections could lead to multi-path problems, which are not investigated in this paper. The quantification of multi-path is what inhibits our analysis, since this non-trivial account must be accompanied with a non-existing tool which is the spike propagation tracking inside neuron networks. Otherwise, only approximations to multi-path quantification could be used, but that is also not yet found by the authors. The simulator script was then modified slightly so that, after the loading of each topology (but before the running of the simulation), some parameters of the communication link were forcibly varied. These parameters included the link delay and multiplicative gain (weight), the distance between the nodes, as well as the interval, delay, weight, and symbol probability of the stimulus. These variations were output to a separate “metadata” file for each simulation such that they could be used in the analysis to find correlations in the data. After this, the network was simulated with a simulation length of 1000 ms. This length allows enough of a collection of neuronal data, which typically presents an average of 10× full cycles of typical neuronal response to stimulation step current with <100 ms.

### 2.4. Cell Classification

As previously mentioned, the Linear-Nonlinear-Poisson (LNP) cascade model can be used to model and characterise the response of a neural cell. Generally, this is computed using the spike-triggered-average (STA) of the stimulus sequence; however, this approach is more appropriate when dealing with external stimuli such as a spatio-temporal screen, discussed in [22]. For this reason, the FIR-filter estimation method described in Equation (1) was used to reduce the dimensionality of the measured features. To investigate the objective classification of biological neuronal structures and to train the support vector machine (SVM) and decision tree classifiers, Matlab was used for its community-backed *Classification Learner* tool. This is a tool that takes a dataset as input requests the specification of the features and classes in the dataset, and then quickly trains a number of different classifiers against the data, reporting the individual classifier accuracy, confusion matrix, and receiver-operating-characteristic (ROC) curve. This tool is therefore very useful for quickly getting an idea of how the different forms of classifiers are dealing with the given features and classes. We also trained random forest and artificial neural network classifiers using RapidMiner, which offers a powerful environment for developing data processing and machine learning models besides those included in the Matlab tool. We have previously discussed how each of the cells in the dataset has been classified by the BBP; that is, each cell has an associated layer, m-type, and e-type. As there are over 1000 individual cell models, it is unlikely that a classifier will be capable of gaining any form of functional accuracy in directly classifying the exact cell type. For this reason, the classification of a given cell was broken down into the classification of each of its constituent components, i.e., three separate classifiers were trained to estimate the overall cell type. The first classifier estimates the layer to which the cell belongs based on the filter coefficients extracted through Equation (1). The second classifier estimates the e-type based on the filter coefficients as well as the output of the first classifier (the layer estimation). The final classifier estimates the m-type based on the filter coefficients, the estimated layer, and the estimated e-type. In this format, each classifier is only estimated against 5, 11, and 24 classes, respectively. The other benefit of this approach is that the output of one classifier can be fed into the next, which allows it to take into account the associated probability of one class leading to another. With this approach, we can interchangeably classify multiple variables from the same trained data.

## 3. Results

### 3.1. Communication Features

#### 3.1.1. Delay Estimation

**Figure 4:**
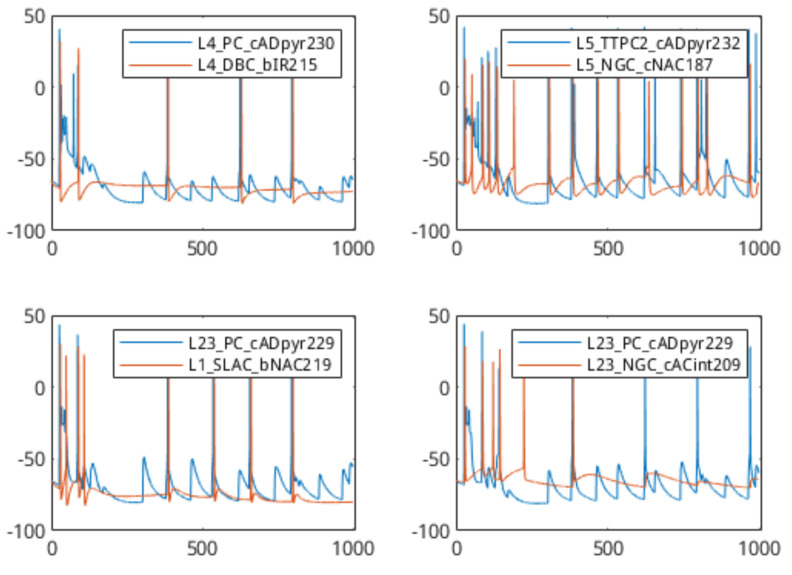
Comparison of 2−cell network outputs. Head-cell in black, tail-cell in orange. Cell-types shown in each subplot’s legend.

The analysis of the communication network details of a synaptic connection was done through the simulation of a set of simple 2-cell networks (*n* = 22,500). This results in the production of sets of voltage data for each simulation, one from the head-cell and one from the tail-cell. As the output of one cell is acting as the stimulus to the other, the spike trains from the simulations tend to be correlated. The output plots from a few of these simulations are shown in Figure 4. In these sample plots, the source–destination spike correlation is clear, as a spike in the pre-synaptic cell often results in a spike in the post-synaptic cell. In addition, the plots indicate a slight delay between the source and event spikes.

**Figure 5:**
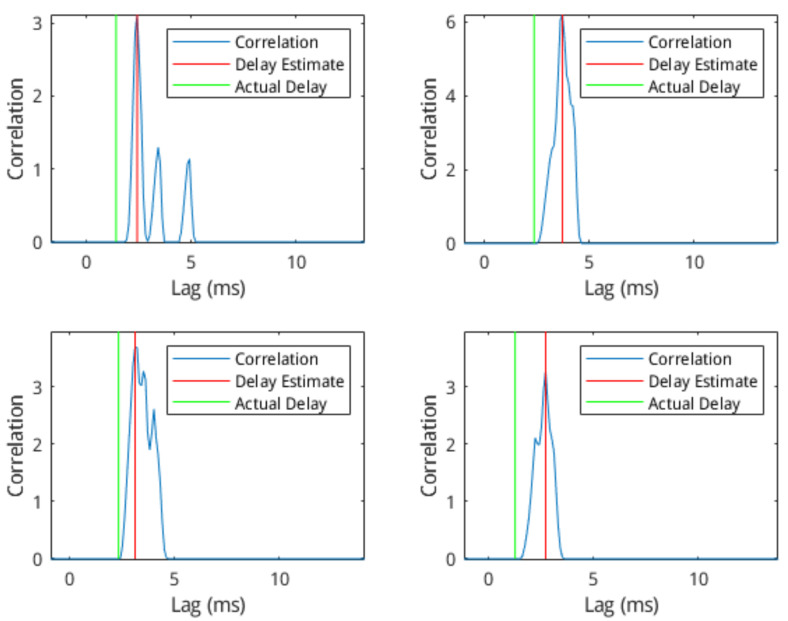
Comparison of 2-cell cross-correlation with delay estimate. Cross-correlation shown in black, estimated delay as a vertical red line, actual delay as a vertical green line.

**Figure 6:**
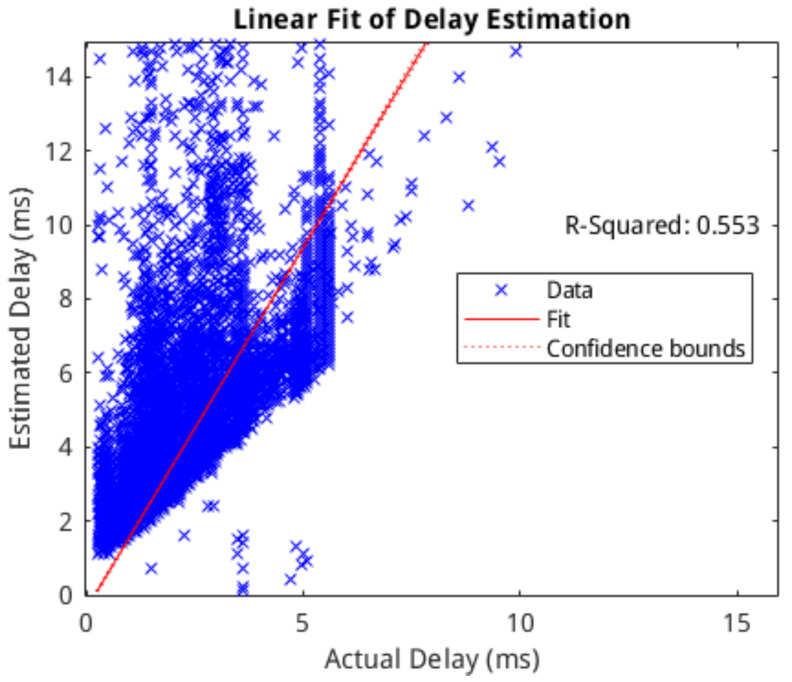
Correlation of delay estimate vs. actual delay with least-squares linear model and associated R-squared score.

Figure 5 shows a number of plots representing this delay estimation. Each subplot shows the cross-correlation of the two signals at a number of lag values from −12 ms to +12 ms, along with the location of the estimated link delay (i.e., the location of the first positive peak) and the location of the actual link delay (as specified by the *delay* parameter). We applied the delay estimation method against all the simulated networks, and the correlation between the estimated delay and the actual delay was analysed. We fit the linear model to the data to determine the correlation between the estimation and the actual delay. A scatter-plot of the estimation and actual delay is shown in Figure 6 along with the least-squares linear fit and associated R-squared score. The mean-squared error of this linear model was 1.0881 ms. These results not only validate our delay estimator but demonstrate its advantage as compared to existing morphological inference structures that are far more complex than the presented model. For example, neuron microcircuits would be comprised of tens of cells.

#### 3.1.2. Entropy and Mutual Information

**Figure 7:**
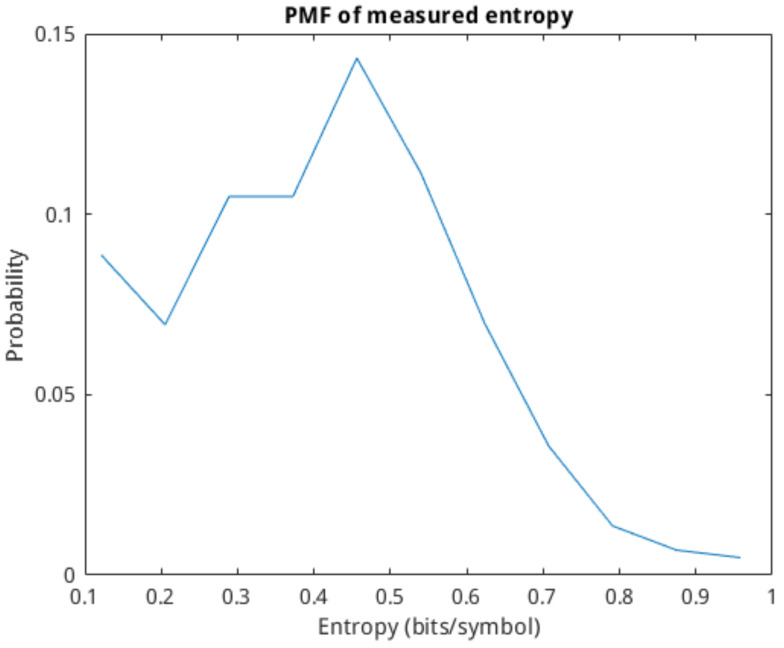
PMF of calculated single-cell entropy based on output spike trains.

**Figure 8:**
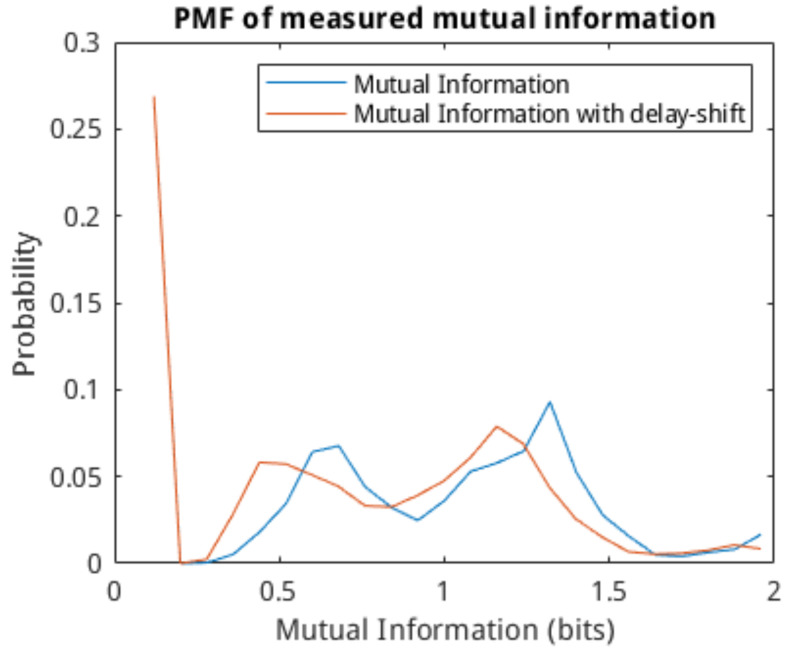
PMF plots of calculated mutual information for the measured spike-train (black) and the delay-estimate shifted spike-train (orange).

As discussed previously, the entropy of the cells is calculated by discretising the spike train and encoding each spike as a binary symbol. Using a symbol sequence, it is then possible to apply the definition of mutual information in Equation (3) to determine the entropy of the individual cells and the mutual information of the cell-to-cell connection. Here, we apply the previously introduced delay estimation model that allows us to shift the spike train of the tail-cell such that the spike–response in that cell becomes virtually instantaneous. This is done to investigate whether or not adjusting the delay in the link will affect the mutual information calculation. The probability mass function (PMF) of the calculated single-cell entropy is shown in Figure 7. The majority of the cells have an entropy of below 0.4 bits/symbol. The PMF of the calculated mutual information is shown in Figure 8. We can see that the mutual information of the networks is well-distributed, with a mean of around 0.5 bits. The effect of shifting the spike-train based on the delay estimate has little to no effect on the distribution of the mutual information since the spike delay variability is low with the used cells. We conclude that entropy and mutual information should be used in parallel for the training of the classification models since they measure different aspects of the communication of neurons.

### 3.2. 2-Cell Classification and Model Training

**Figure 9:**
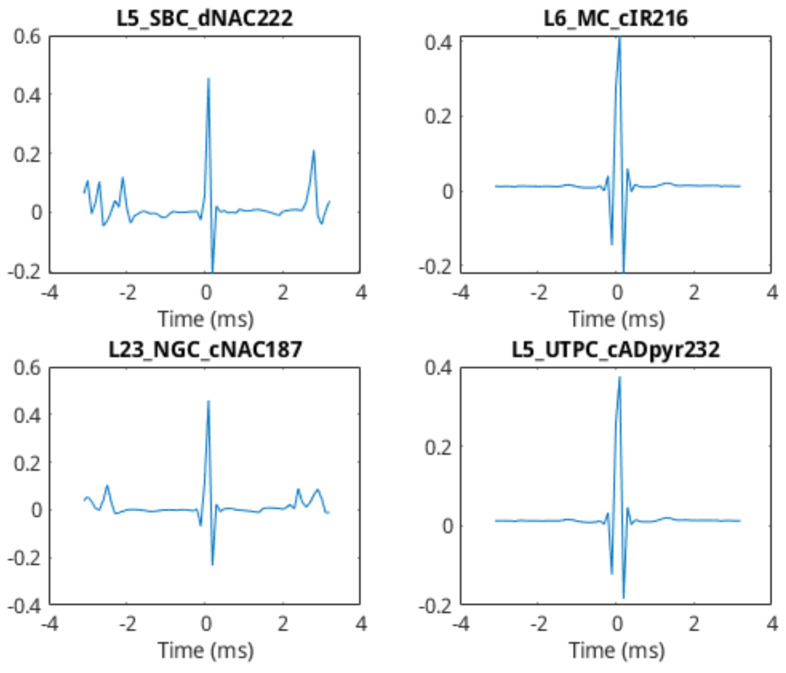
Sample impulse−response characterisation of different cells.

In Figure 4, a number of spike-trains from different cell types were shown. Referring back to that, it is clear that different cells respond differently to the same stimulus, with the output spike train differing in intensity (frequency of spikes) as well as in the “settle-down” period after a spike was triggered (i.e., the fall-time response of the membrane voltage). It is these differences that we attempt to characterise through the use of the linear-filter portion of the Linear-Nonlinear-Poisson (LNP) cascade model. The first step in this process is to simulate some 2-cell networks again to generate test data. In this instance, the head-cell acts only as a stimulus generator, and we treat the soma-membrane potential of this cell as the “input” voltage to the linear system described in Section 2.2. A large number of networks was generated (*n* = 30,000) to produce a dataset of sufficient size for classifier training. Following simulation and before training the classifiers, the filter estimation step described in Equation (1) was applied to extract the filter coefficients as features from the cell-response data. Figure 9 shows the filter coefficients (FIR impulse response) of four different cells, with a filter-order of 64. It is clear from this diagram that the impulse-response estimation of the neuronal cell is capable of differentiating between the cell types. Using these estimated filter coefficients, we are now able to begin training the classifiers.

Using the *Classification Learner* tool in Matlab, we can train and compare a set of different classifiers, inspecting the accuracy, confusion matrix, and receiver-operating characteristic (ROC) curve for each. The first classifier to investigate is the layer predictor, which takes the estimated filter coefficients as input, and attempts to estimate what layer the observed cell belongs to. We use this tool to train and analyse an SVM and decision tree classification systems, while we use *RapidMiner* to train the random forest and artificial neural network classifiers. We kept the configuration of the classification as standard, since optimising the classification tools would possibly interfere with the quantification of the effect of the communication metrics as a feature for classification. For further reference, visit [32,33] for detailed configuration information. The performance of the classifiers is shown in Table 1, Table 2 and Table 3. In each table, we compare the performance of the different classification algorithms (Decision Tree, SVM, etc.) in the classification of the separate cell sub-group types (layer, m-type, e-type). For each classifier, we state the corresponding accuracy. The accuracy is calculated as the ratio of correct estimations versus the total number of estimations. We also state the “factor of improvement” of the classifier. This is calculated as the factor by which the classifier improves over the equivalent accuracy of random guessing in the classification space. For example, with five layer classes, the equivalent “random guess” accuracy is 20%, and so a trained classifier with an accuracy of 40% would have a factor of improvement of 2. Table 1 shows the performance of the classifiers in the prediction of cell layer-type, Table 2 for m-type prediction, and Table 3 for e-type prediction. In every case, the SVM classifier has the highest accuracy, with the Neural Network being slightly behind. Of the tree-based classifiers, interestingly, the decision tree based classifier consistently has a higher accuracy than the random forest classifier, despite the latter being a variant of the former. That is based on the construction of the trees used in both techniques, and the simple data structures used for training, which leads to more efficient tree structures based on deterministic approaches (decision trees) as compared to probabilities approaches (random forests).

Another set of metrics that are used to quantify the performance of a classifier are the class precision and the class recall. The class recall is calculated per-class as the ratio of correct predictions of a class versus the number of observations of that class. The class precision is again calculated per-class as the ratio of correct predictions of a class versus the overall number of estimations per class. As these metrics identify the performance of the model on a class-by-class basis, they result in a large number of individual values to compare. When we consider that we are comparing three estimator groups with a relatively high number of classes per group (five for layer estimator, 25 for m-type, and 14 for e-type), as well as the fact that each estimator group must be compared against four different classification algorithms, it becomes difficult to critically compare the performance of each individual class. In general, however, when analysing such metrics for the single classifier, a *confusion matrix* is used. The confusion matrix of the SVM-based layer estimator is shown in Table 4. The confusion matrix tabulates the estimations of a given classifier as a grid of “True Class” versus “Predicted Class”. In this way, it can show how often an observation of a given class is estimated as belonging to some other class. As such, the diagonal (shown as green in the confusion matrix figure) shows the proportion of the correct predictions in the validation set (where true class equals predicted class). The right-hand bar shows the proportional difference of the *true positive rate* and the *false negative rate*. The true-positive rate is equivalent to the class recall. While the confusion matrices for every single classifier investigated in this study are not included, the relative proportions between them tend to be similar (i.e., very high prediction accuracy between layer 1 and layer 6, with reduced accuracy in the intermediate layers).

**Figure 10:**
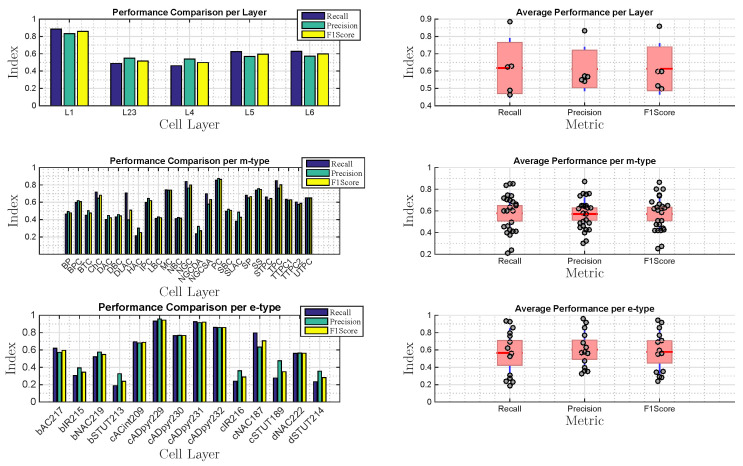
Results for the comparison of different types of cell layers, m-types and e-types considering recall, precision and F1 score performance. Red lines represent the mean values and the blue lines are the error bars.

Performance comparison between layers, m-type as well as e-type present also variability for metrics such as recall, precision and F1 score. In Figure 10, while we show an average close to 0.6 of all metrics for all layers, we can observe that classifying cells in layer one is often easier. Many reasons might be thought for that, but they all boil down to the easy propagation of spike signals in that layer. The variability in the performance is higher when considering the comparison of m-types and e-types. Our results show that the link between structure and properties of neurons presents different levels of communication, which can be classified. While in some cell types this detection is still poor (like NGCDA m-type or bSTUT213 e-type), in other cells, it presents really high classification performance (like PC m-type or cADpyr229 e-type).

The truth-table for all neuron types can be found in Appendix A. The variability in the presented results can be attributed to the spike variability for cell-cell communication as well as fine-tuning of the machine learning techniques used. A future solution to this problem could be found not only by adjusting the machine learning configuration for each type of cell, but also looking at different types of machine learning techniques and their combination. Even though this seems an arduous task, investigating these proposed solutions will bring tremendous benefits in detecting novel patterns of communication as well as structure in the brain that have not been identified to date.

The ROC receiver operating characteristic curve are presented in Appendix B to show the generality, in terms of performance, for the SVM classifier.

#### Network Tomography for Cellular Classification

**Figure 11:**
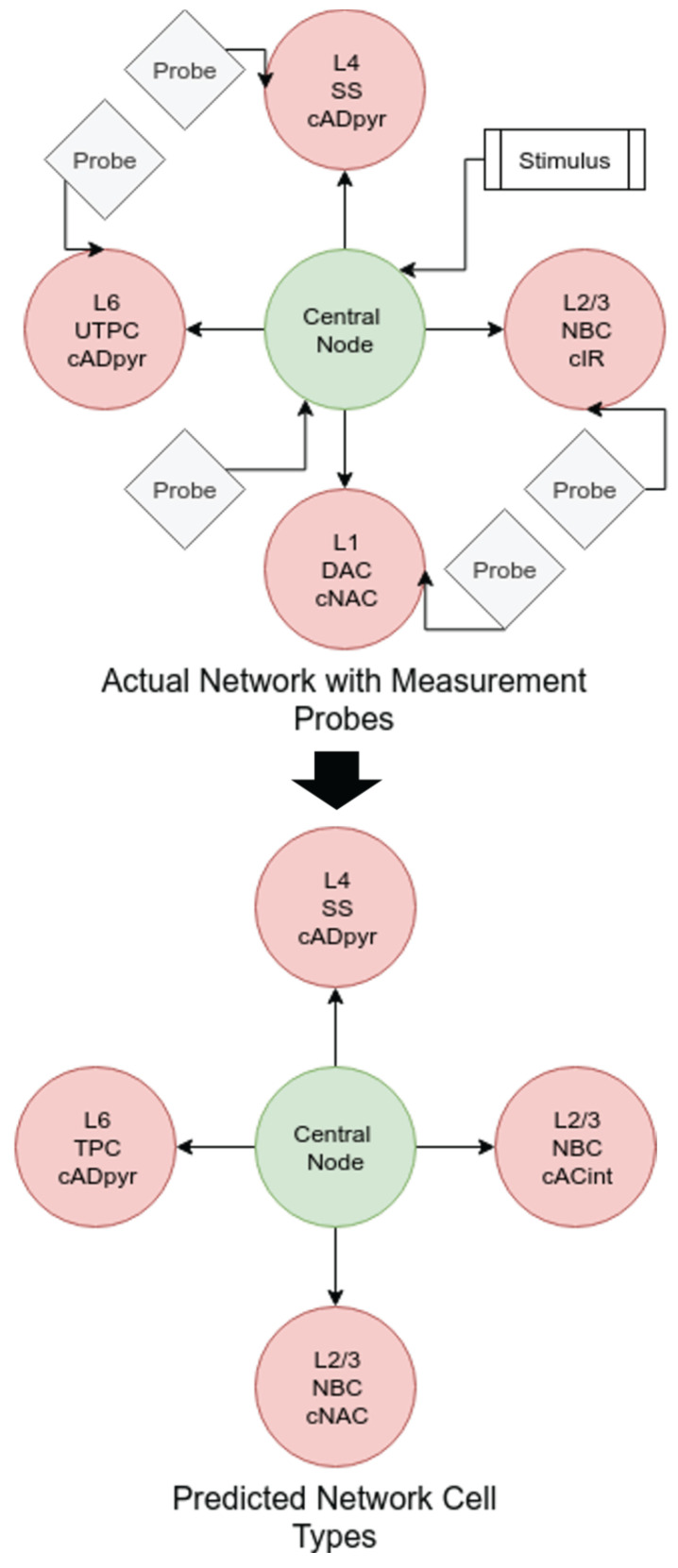
Sample reconstruction of network.

As the SVM classifier had the best performance in comparison to the other classification models, this is the model that was applied in the reconstruction of the 4-leaf star networks. The process here was similar to the previous experiments: several unique networks were generated, simulated, and their data-points measured and analysed. In this case, the networks generated were of the 4-leaf star topology type previously discussed. The central node was constrained to be of the same cell type used in the training of the models (layer 1, DAC m-type, bNAC e-type), while the star cells were varied. For each measurement from the soma-membrane of a star node, the characteristic filter was estimated using the same process discussed previously, and the filter coefficients were passed through the pre-trained classifiers. The performance results of the topology reconstruction are shown in Table 5. Here, we tabulate the accuracy of the classifier chain in estimating layer, m-type, and e-type groups, as well as the “whole-cell” classification accuracy. We define whole-cell accuracy as the cases where all three sub-groups were correctly estimated. The factor of improvement for the whole-cell prediction is significantly higher than for any other group, while the sub-group accuracy is quite similar to that in the 2-cell networks. Figure 11 shows a sample reconstruction of a 4-leaf network from probe measurements. The top part of the figure represents the network as it was simulated, with probes at the network endpoints, probe in the central node, and a stimulus on the central node, along with the actual cell types of the leaf nodes. The bottom part of the figure shows the reconstructed topology based on the cell-type estimations from the SVM classifier chain. The top figure is the original network topology, and the bottom one is the predicted network topology.

## 4. Discussion

The presented objective classification of cells from neural communication data shows promising results, with relatively high accuracy considering the high number of classes that the models were estimating. The SVM-based classifier showed the highest level of accuracy, with the artificial neural network being close behind in performance. This was surprising, as neural networks are widely used for high-order classification problems. It is possible, however, that the close level of accuracy between the two is indicative of the limit of the features on which we are predicting and that the level of information that the features hold does not allow for accuracy above this level. The classifier with the highest performance in all cases was the SVM-based model. In classifying the layer type, the SVM model was capable of achieving 62.5% accuracy, a factor of improvement of 3.13 over random guessing. By looking at the confusion matrix for this classifier in Table 4, we can see that the classifier has very good performance in classifying between layer 1 and layer 6 with close to 100% accuracy between these classes, where only 1% of layer 6 cells were predicted as layer 1, and less than 1% of layer 1 cells were predicted as layer 6. The overall accuracy decreases as the intermediate layers are added, with the worst-performing component incorrectly predicting a layer 4 cell as layer 5 for 21% of the time. It is evident, therefore, that the highest degree of separation using the FIR-filter estimation is between layer 1 and layer 6. In the reconstruction of the 4-leaf topology through endpoint measurements using the trained SVM classifier, the individual sub-group accuracy values tend to reflect those of the 2-cell networks with a layer-estimation accuracy of 61.82%, an m-type accuracy of 56.34%, and an e-type accuracy of 64.62% representing a factor of improvement of 3.09, 14.09, and 9.05, respectively. The interesting result in this investigation, however, is the whole-cell estimation (where each of the individual sub-group estimators was correct) with an overall accuracy of 36.23% and a huge factor of improvement of 634.5. Several areas could be worked on in the future to extend the presented investigation. One of them is the variation of the central/stimulus cell. In practice, many different cell types may interconnect and so the ability to classify a cell regardless of the presynaptic cell type is important. This can create a large set of dynamics that must also be captured by the classification model [34]. Another consideration for future work would be to assume multi-path networks enabling classification and inference of higher complexity cortical structures. In practice, a given cell may be stimulated by a number of presynaptic cells. This can be characterised using the LNP model by using multiple linear filters and computing the output based on a combination of the individual coefficients [22]. In our study, we used only excitatory presynaptic cells to generate measurements with a large number of characterising spikes. Additionally, metrics that rely on molecular information can also be added [35], which can potentially classify cell types over time and detecting as well as predicting disease states in the tissue [36].

## 5. Conclusions

To contribute to advancements in objective morphological classification of neurons, we present a technique that uses information network tomography to classify and infer the neurons’ morphological, topological and electrical types and networks in the somatosensory cortex of the brain. We use information transfer metrics as well as extend existing signal processing tools to accommodate larger cortical structures in our prediction analysis, such as small-world topologies. With an average accuracy of around 70% across the layer, m-type, and e-type class groups, the SVM-based classifier outperforms decision tree, random forest, and even artificial neural network classifiers. We have also shown that the trained classifier can then be applied in the reconstruction of the cell-types in various forms of the 4-leaf star topology, estimating the cell-type of each leaf node to a promising degree of accuracy. Besides the promising performance of our proposed system, it is clear that more research is required to improve the robustness of this form of classification to any usable degree. While the investigations carried out in this study were preliminary regarding the extremely wide scope of neurology, molecular communication, network tomography, and information theory, we provided evidence that the proposed approach may be a promising starting point for the robust and reliable in-vivo detailed characterisation of neuronal structures using future miniaturised implantable devices.

## Figures and Tables

**Table 1:** Performance of different algorithms at classifying cell-layer (5 classes, 20% equivalent random guessing accuracy).

Classifier	Decision Tree	Random Forest	SVM	NN
Accuracy	67.30%	53.26%	80.60%	81.31%
FactorImprovement	3.365	2.663	4.03	4.0655

**Table 2:** Performance of different algorithms at classifying cell m-type (25 classes, 4% equivalent random guessing accuracy).

Classifier	Decision Tree	Random Forest	SVM	NN
Accuracy	52.10%	52.93%	74.20%	69.62%
FactorImprovement	13.025	13.2325	18.55	17.405

**Table 3:** Performance of different algorithms at classifying cell e-type (14 classes, 7.143% equivalent random guessing accuracy).

Classifier	Decision Tree	Random Forest	SVM	NN
Accuracy	54.90%	49.06%	64.70%	60.59%
FactorImprovement	7.685846283	6.868262635	9.057818844	8.482430351

**Table 4:** Confusion Matrix of SVM layer−classifier. The colors represent the level of performance of the classification task per cortical layer. Green represent strong performance level, and red represent weak performance level. The darkness of the color represents its value, being the darkest the highest values.

		Predicted Class					True Pos.	False Neg.
		L1	L2/3	L4	L5	L6		
True Class	L1	87.1%	9.3%	0.7%	2.4%	0.5%	87.1%	12.9%
	L2/3	5.8%	84.9%	7.9%	0.5%	0.9%	84.9%	15.1%
	L4	0.3%	5.9%	87.7%	4.4%	1.7%	87.7%	12.3%
	L5	0.9%	0.2%	5.2%	77.7%	15.9%	77.7%	22.3%
	L6	0.6%	0.2%	1.0%	32.5%	65.7%	65.7%	34.3%

**Table 5:** Performance of 4-leaf star topology reconstruction.

Prediction	Layer	m-Type	e-Type	Whole-Cell
# classes	5	25	14	1750
Equiv. random guess accuracy	20.0%	4.0%	7.143%	0.0571%
Classifieraccuracy	61.82%	56.34%	64.62%	36.23%
FactorImprovement	3.091	14.085	9.05	634.5

**Figure A1:**
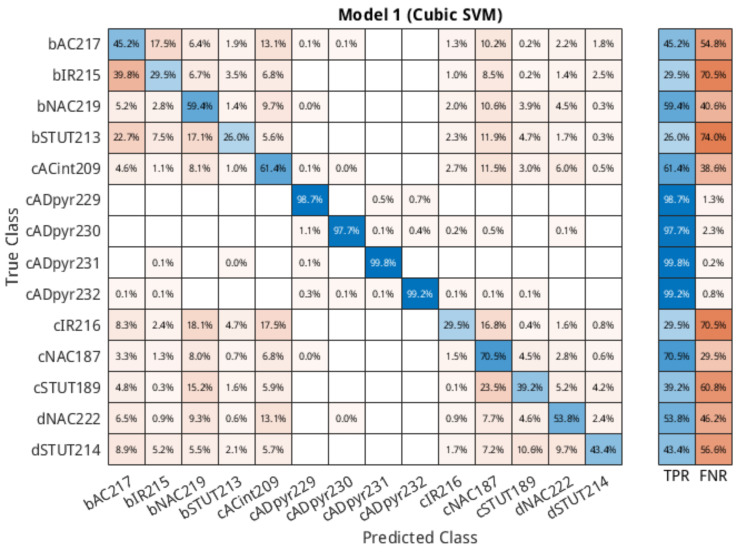
E-type confusion matrix. The colors represent the level of performance of the classification task per cortical layer. Blue represent strong performance level, and red represent weak performance level. The darkness of the color represents its value, being the darkest the highest values.

**Figure A2:**
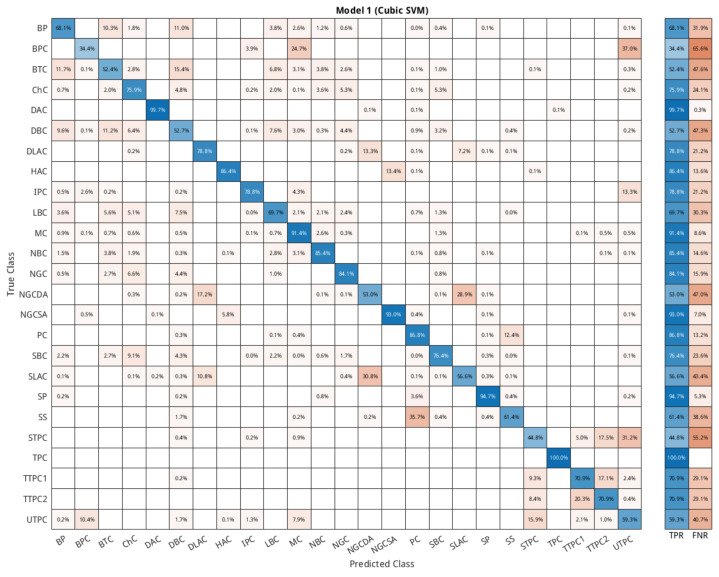
M-type confusion matrix. The colors represent the level of performance of the classification task per cortical layer. Blue represent strong performance level, and red represent weak performance level. The darkness of the color represents its value, being the darkest the highest values.

**Figure A3:**
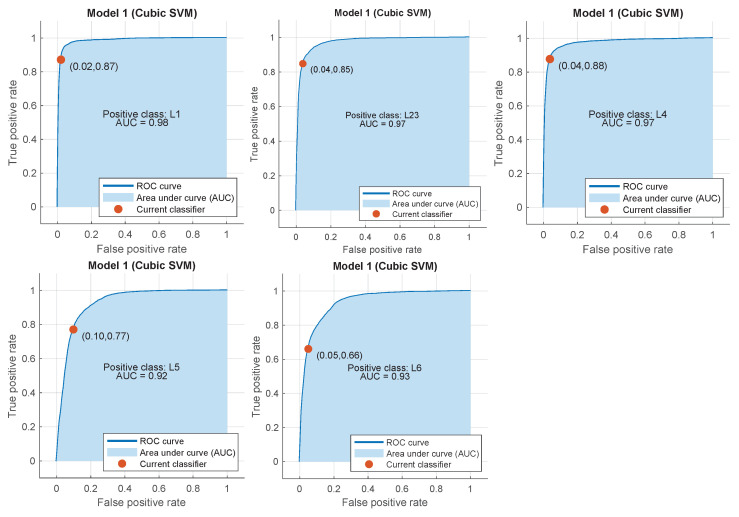
ROC curves.

## Data Availability

The datasets generated and analysed for this study can be found in the github https://doi.org/10.5281/zenodo.4009922 and https://doi.org/10.5281/zenodo.4009917 (accessed on 21 July 2022).

## Appendix A. E-Type and M-Type Confusion Matrix

In this appendix, we provide the confusion matrix for the e-type and m- type classification individually. Please find them below. This is depicted in Figure A1 and Figure A2, respectively.

## Appendix B. ROC Curves

In this appendix, we provide the ROC curves (receiver operating characteristic curve), for our best performing classifier to give an insight of the generality of our models. This is depicted in Figure A3. We only add results of the layers that were classified, as there would be several graphs to include for each layer type which is unfeasible to include in the paper.

## References

[1] Markram H., Muller E., Ramaswamy S., Reimann M.W., Abdellah M., Sanchez C.A., Ailamaki A., Alonso-Nanclares L., Antille N., Arsever S. (2015). Reconstruction and Simulation of Neocortical Microcircuitry. Cell.

[2] Kanari L., Ramaswamy S., Shi Y., Morand S., Meystre J., Perin R., Abdellah M., Wang Y., Hess K., Markram H. (2019). Objective morphological classification of neocortical pyramidal cells. Cereb. Cortex.

[3] Vasques X., Vanel L., Villette G., Cif L. (2016). Morphological neuron classification using machine learning. Front. Neuroanat..

[4] Barros M.T., Siljak H., Ekky A., Marchetti N. (2019). A Topology Inference Method of Cortical Neuron Networks Based on Network Tomography and the Internet of Bio-Nano Things. IEEE Netw. Lett..

[5] Balasubramaniam S., Wirdatmadja S.A., Barros M.T., Koucheryavy Y., Stachowiak M., Jornet J.M. (2018). Wireless communications for optogenetics-based brain stimulation: Present technology and future challenges. IEEE Commun. Mag..

[6] DeFelipe J., López-Cruz P.L., Benavides-Piccione R., Bielza C., Larrañaga P., Anderson S., Burkhalter A., Cauli B., Fairén A., Feldmeyer D. (2013). New insights into the classification and nomenclature of cortical GABAergic interneurons. Nat. Rev. Neurosci..

[7] Deitcher Y., Eyal G., Kanari L., Verhoog M.B., Atenekeng Kahou G.A., Mansvelder H.D., De Kock C.P., Segev I. (2017). Comprehensive morpho-electrotonic analysis shows 2 distinct classes of L2 and L3 pyramidal neurons in human temporal cortex. Cereb. Cortex.

[8] Barros M.T. (2017). Ca^2+^-signaling-based molecular communication systems: Design and future research directions. Nano Commun. Netw..

[9] Moioli R.C., Nardelli P.H., Barros M.T., Saad W., Hekmatmanesh A., Silva P.E.G., de Sena A.S., Dzaferagic M., Siljak H., Van Leekwijck W. (2021). Neurosciences and wireless networks: The potential of brain-type communications and their applications. IEEE Commun. Surv. Tutor..

[10] Gal E., London M., Globerson A., Ramaswamy S., Reimann M.W., Muller E., Markram H., Segev I. (2017). Rich cell-type-specific network topology in neocortical microcircuitry. Nat. Neurosci..

[11] Yang R., Sala F., Bogdan P. (2021). Hidden network generating rules from partially observed complex networks. Commun. Phys..

[12] Xiao X., Chen H., Bogdan P. (2021). Deciphering the generating rules and functionalities of complex networks. Sci. Rep..

[13] Yin C., Xiao X., Balaban V., Kandel M.E., Lee Y.J., Popescu G., Bogdan P. (2020). Network science characteristics of brain-derived neuronal cultures deciphered from quantitative phase imaging data. Sci. Rep..

[14] Akan O.B., Ramezani H., Khan T., Abbasi N.A., Kuscu M. (2016). Fundamentals of molecular information and communication science. Proc. IEEE.

[15] Ramezani H., Akan O.B. (2018). Impacts of spike shape variations on synaptic communication. IEEE Trans. Nanobiosci..

[16] Balevi E., Akan O.B. (2013). A physical channel model for nanoscale neuro-spike communications. IEEE Trans. Commun..

[17] Barros M.T. (2018). Capacity of the hierarchical multi-layered cortical microcircuit communication channel. Proceedings of the 5th ACM International Conference on Nanoscale Computing and Communication.

[18] Veletić M., Floor P.A., Babić Z., Balasingham I. (2016). Peer-to-peer communication in neuronal nano-network. IEEE Trans. Commun..

[19] Strong S.P., Koberle R., van Steveninck R.R.D.R., Bialek W. (1998). Entropy and information in neural spike trains. Phys. Rev. Lett..

[20] Glaser J.I., Benjamin A.S., Farhoodi R., Kording K.P. (2019). The roles of supervised machine learning in systems neuroscience. Prog. Neurobiol..

[21] Herfurth T., Tchumatchenko T. (2019). Quantifying encoding redundancy induced by rate correlations in Poisson neurons. Phys. Rev. E.

[22] Chichilnisky E. (2001). A simple white noise analysis of neuronal light responses. Netw. Comput. Neural Syst..

[23] Smith J.O. (1983). Techniques for Digital Filter Design and System Identification with Application to the Violin. Ph.D. Thesis.

[24] Ljung L., Söderström T. (1983). Theory and Practice of Recursive Identification.

[25] Zbili M., Debanne D. (2019). Past and future of analog-digital modulation of synaptic transmission. Front. Cell. Neurosci..

[26] Bassett D.S., Bullmore E.T. (2017). Small-world brain networks revisited. Neuroscientist.

[27] Coates M., Hero A., Nowak R., Yu B. (2002). Internet tomography. IEEE Signal Process. Mag..

[28] Chen A., Cao J., Bu T. (2010). Network Tomography: Identifiability and Fourier Domain Estimation. IEEE Trans. Signal Process..

[29] Hines M.L., Carnevale N.T. (1997). The NEURON simulation environment. Neural Comput..

[30] (2016). Project, Blue Brain NMC Portal. https://bbp.epfl.ch/nmc-portal/welcome.html.

[31] Ramaswamy S., Courcol J.D., Abdellah M., Adaszewski S.R., Antille N., Arsever S., Atenekeng G., Bilgili A., Brukau Y., Chalimourda A. (2015). The neocortical microcircuit collaboration portal: A resource for rat somatosensory cortex. Front. Neural Circuits.

[32] Mullen P. (2019). Neurpy Library. https://zenodo.org/record/4009922#.YxHBUHZBxPY.

[33] Mullen P. (2019). Research Release Code. https://zenodo.org/record/4009917#.YxHBjXZBxPY.

[34] Ofer N., Shefi O., Yaari G. (2019). Axonal tree morphology and signal propagation dynamics improve interneuron classification. bioRxiv.

[35] Barros M., Dey S. (2018). Feed-forward and feedback control in astrocytes for Ca^2+^-based molecular communications nanonetworks. IEEE/ACM Trans. Comput. Biol. Bioinform..

[36] Barros M.T., Silva W., Regis C.D.M. (2018). The multi-scale impact of the Alzheimer’s disease on the topology diversity of astrocytes molecular communications nanonetworks. IEEE Access.

